# Evidence of validity of the Competence Scale of Actions of Nurses in Emergencies^*^

**DOI:** 10.1590/1518-8345.2814.3128

**Published:** 2019-03-18

**Authors:** Flávia Lilalva de Holanda, Celina Castagnari Marra, Isabel Cristina Kowal Olm Cunha

**Affiliations:** 1 Faculdade de Medicina de Jundiaí, Jundiaí, SP, Brasil.; 2 Universidade Federal de São Paulo, Escola Paulista de Enfermagem, São Paulo, SP, Brasil .

**Keywords:** Employee Performance Appraisal, Professional Competence, Behavior Rating Scale, Nursing in Emergency, Psychometrics, Validity of Tests, Avaliação de Desempenho Profissional, Competência Profissional, Escala de Avaliação de Comportamento, Enfermagem em Emergência, Psicometria, Validade dos Testes, Evaluación del Rendimiento de Empleados, Competencia Profesional, Escala de Evaluación de la Conducta, Enfermería en Urgencia, Psicometría, Validez de las Pruebas

## Abstract

**Objective:**

to evaluate the validity of the Competence Scale of Actions of Nurses in Emergencies based on internal structure, internal consistency, and external criteria.

**Methods:**

methodological study to verify new evidence of validity of the Scale, with contents previously validated. The Scale has 81 measurable actions at five levels of competence and can be applied both for self- and/or hetero-evaluation.

**Results:**

one hundred and forty seven nursing assistants and 41 managers from the five regions of Brazil participated in the study. They were linked to mobile prehospital emergency service, fixed prehospital emergency service, or hospital emergencies. Dimensionality was evidenced by exploratory factorial analysis of the 81 items, pointing out seven factors that explained 66.5% of the total data variance. Cronbach’s alpha ranged from 0.79 to 0.98. The Kaiser-Meyer-Olkin 0.988 indicated that the correlations between the items were significant. In the external criterion, Pearson’s correlations between hetero-evaluation competence scores and the manager’s subjective classification were significant (*p* < 0.001), as well as differences in the means of these competencies by criterion group. In addition, scores by characteristics were evaluated to detect statistically different means.

**Conclusion:**

through the adopted Statistical Procedures, with multi-methods and multi-informants, different psychometric properties were analyzed. A summary of evidence was generated showing that the Scale is valid and reliable.

## RESUMO

**Objetivo:**

avaliar evidências de validade da Escala de Competências das Ações do Enfermeiro em Emergências com base na estrutura interna, na consistência interna e no critério externo.

**Métodos:**

estudo metodológico para verificação de novas evidências de validade da Escala, com conteúdos previamente validados. A Escala com 81 ações mensuráveis em cinco níveis de competências pode ser realizada tanto para o enfermeiro se autoavaliar como outros o avaliarem.

**Resultados:**

participaram 407 enfermeiros assistenciais e 41 gestores das cinco regiões do Brasil, atuantes em emergências pré-hospitalar móvel, fixa ou hospitalar. A dimensionalidade foi evidenciada mediante análise fatorial exploratória dos 81 itens, apontando sete fatores que explicaram 66,5% da variância total dos dados. O alfa de Cronbach variou de 0,79 a 0,98. O Kaiser- Meyer-Olkin 0,988 indicou que as correlações entre os itens foram significantes. No critério externo, correlações de Pearson entre escores de competências de heteroavaliação e classificação subjetiva do gestor foram significantes (*p*<0,001), bem como diferenças das médias dessas competências por grupo critério. Adicionalmente, avaliaram-se escores por características, verificando-se médias estatisticamente distintas.

**Conclusão:**

por meio dos Procedimentos Estatísticos adotados, com multimétodos e multi-informantes, analisaram-se diferentes propriedades psicométricas, gerando um sumário de evidências, demostrando que a Escala é válida e confiável.

## RESUMEN

**Objetivo:**

evaluar evidencias de validez de la Escala de Competencias de las Acciones del Enfermero en Emergencias con base en la estructura interna, en la consistencia interna y en el criterio externo.

**Métodos:**

estudio metodológico para verificación de nuevas evidencias de validez de la Escala, con contenidos previamente validados. La Escala con 81 acciones mensurables en cinco niveles de competencias puede ser realizada tanto para que el enfermero se autoevalúe como otros lo evalúen.

**Resultados:**

participaron 407 enfermeros asistenciales y 41 gestores de las cinco regiones de Brasil, actuantes en emergencias pre-hospitalaria móvil, fija o hospitalaria. La dimensionalidad fue evidenciada mediante análisis factorial exploratoria de los 81 ítems, apuntando siete factores que explicaron 66,5% de la variancia total de los datos. El alfa de Cronbach varió de 0,79 a 0,98. El Kaiser- Meyer-Olkin 0,988 indicó que las correlaciones entre los ítems fueron significantes. En el criterio externo, correlaciones de Pearson entre puntajes de competencias de heteroevaluación y clasificación subjetiva del gestor fueron significantes (*p*<0,001), así como diferencias de las medias de esas competencias por grupo criterio. Adicionalmente, se evaluaron puntajes por características, verificándose medias estadísticamente distintas.

**Conclusión:**

por medio de los Procedimientos Estadísticos adoptados, con multimétodos y multiinformantes, se analizaron diferentes propiedades psicométricas, generando un sumario de evidencias, demostrando que la Escala es válida y confiable.

## Introduction

In the process of evaluating professional competence, several elements are taken into consideration in the creation of a research tool capable of extracting from the respondents the best response to the alternatives offered. The availability of valid tools for assessing the workforce is essential to measure the actual competencies as well as to identify the area of practice to be developed^1^. 

In Psychometrics, the concept of validity can be generically defined as the degree to which theoretical-empirical evidence supports inferences and interpretations about people’s psychological characteristics. This is done based on behaviors observed/measured by a measuring instrument, always considering the relevance and usefulness of the uses proposed in certain contexts^2^ ^-^ ^5^. 

To date, there are no Brazilian studies published on the creation of technology capable of measuring the professional competence of nurses working in emergencies. As there is no instrument available, it is essential to create one to measure the professional competence of nurses who act in emergencies based on the profile of the professional, the client, the institution and the public policy of the Brazilian emergency care. 

The creation of the Instrument for Assessment of the Pofessional Competence of Nurses in Emergencies used psychometrics as reference and involved three stages, namely: Theoretical Procedures, Empirical/Experimental Procedures, and Analytical/Statistical Procedures^6^. Firstly, the Theoretical Procedures proposed a Professional Competence Matrix (PCM) with two types of compentencies to be considered: Basic Competencies (BC) or Associated Competencies (AC), as well as the number of compentencies in each; then to, they were constitutively defined^7^. From the eight basic competencies (BCs) and the 32 Associated Competencies (ACs) indicated on the PCM^7^, the operational definition of 56 attitudes/behaviours was made, represented by Identifying Questions (IQs),which resulted in the Professional CompetenceProfile (PCP)^8^. Then, evidence of validity was verified through the Delphi Technique based on the content of the IQs by nurseswho were *experts* in the theme, with 90% agreement and 98.61 of the Content Validity Index^9^. These 56 IQs were separated into 81 unique actions, composing the Pilot Instrument^10^.

The content of the instrument included personal/professional/academic characterization data, a Scale of Classification the Degree/Level of Professional Competence, three fictitious cases to assess the level of competence, and a spreadsheet with 81 measurable actions in five levels of competencies. This set of measurable actions formed the Competence Scale of Actions of Nurses in Emergencies (CSANE), understood as an instrument capable of measuring the attitudes/behaviors arising from the professional exercise as informed by the nurses or identified by third parties according to the degree/level of competence required/established on a proper scale. It can be used for both self- and hetero-evaluation. 

Self-evaluation consists in the evaluation of nurses of their own attitudes/behaviors inherent to their daily nursing care practices according to the degree/level of competence attributed to each one of them. In turn, hetero-evaluation is performed by a manager to generate an external evaluation of staff nurses working directly with provision of care in their daily routine, according to the degree/level of competence identified from the perspective of this manager.

Although the validity of the content of these attitudes/behaviors is an important source, it is not enough. Demonstrating evidence of validity to use a measuring instrument is a continuous and cumulative process of studies that aggregate a set of scientific evidence^3^ ^-^ ^4^ ^,^ ^6^ ^,^ ^11^ ^-^ ^13^. 

In order to continue the processes of construction, application and creation of a body of evidence of validity, we sought sources of information that strengthened it through Empirical/Experimental Procedures and Analytical/Statistical Procedures^6^. 

In view of the above, the question raised is: Does the Scale created based on the PCM and the PCP have other sources of evidence of validity? To answer this question, we tried to evaluate evidence of validity of the Competence Scale of Actions of Nurses in Emergencies based on internal structure, internal consistency and external criteria, according to Psychometrics. Thus, the study was based on the hypothesis that the professional competences are distributed in a multifactorial structure, that the hetero-evaluations are correlated, and that the competent performance of the subject in a given item is explained by the direction of the answers given as a function of the latent trait. 

## Method

This was a methodological study, part of a larger research started in 2013 at a public university located in the city of São Paulo, Brazil. In the present phase, we used Psychometrics, specifically Empirical and Statistical Procedures^6^, as references to check the behavioral representation of the construct. 

In the Empirical Procedures, the pilot instrument created^10^ was tested to define and calculate the sample and the data collection procedures. As the proposed instrument can be used for self-evaluation of staff nurses, independently of their time working in emergency services, and for evaluation by other professionals, the instrument was tested in a university hospital. The nursing team of this type of hospital is composed of newly trained or non-qualified nurses with or without specialization or who are specializing in emergency care or other areas, characterizing therefore a variable sample. 

After testing it in April 2015, the instrument was adjusted and the sample size was calculated. A minimum of five staff nurses was defined for each of the 81 items, totaling 405 respondents. As for the number of nurse managers, it was not possible to predetermine the number of participants because the exact contingent that would make the hetero-evaluation was not known. This data did not affect the result because the manager could only make the hetero-evaluation of nurses who are under his/her responsibility. 

Therefore, the sample was intentional, consisting of staff nurses and the nurse managers responsible for them. The staff nurses did the self-evaluation, and the managers did the hetero-evaluation of the staff nurses and another subjective hetero-evaluation. These nurses worked in services linked to the Urgency and Emergency Network (UEN), integrated with different points of attention: Hospitals and Mobile and Fixed Prehospital Care Services (PHCS). The choice of these three points was justified by the fact that the care for clients with acute or serious conditions in the Unified Health System (SUS) should be provided in one of these services. 

Twelve reference hospitals in the area, participants of the 1st Cycle of S.O.S. Emergencies were chosen to integrate the sample. These hospitals had emergency services with “door open”, 24-hour service every day of the week, with spontaneous and referenced demand of clinical, pediatric, surgical or traumatic urgency. As for the Mobile Emergency Care Service (SAMU), we chose the bases located in São Paulo because this is the most populous city in the country and with offer of care at different levels of complexity. The The fixed PHCS was the Ambulatory Medical Care (AMC) unit, a service with intermediary complexity that allows integration between primary care, SAMU and hospitals. An AMC unit near a hospital of its reference was chosen. It should be noted that AMC units are equivalent to Emergency Care Units (ECUs) in Brazil, but for political reasons, they were named differently in São Paulo. 

According to Pasquali (2009), the observed score is equal to the true score plus the error^11^. Therefore, the evaluation involving a broader reasoning, combining multimethods and multi-informants, solving inconsistencies, will probably provide more realistic information about the person evaluated. 

Then, the institutions were contacted by telephone calls and electronic mails. One hospital did not return the contact and was excluded from the study.

Data was collected between May 2015 and January 2016 from nurses who agreed to participate in the study by signing the Informed Consent Term (ICT). Nurses on leave, leave and post-graduate students *lato sensu* were excluded.

To collect the data, the researcher used a pre-established routine, following specific steps according to the profile of the institution and the sample: presentation, verbal invitation, thanks, agreement to participate, indication of the collection room, delivery and receipt, of the ICT and of the Professional Competence Instrument. First, the staff nurse and the manager filled out the part on characterization data and evaluated the cases. Then, the self-evaluation or the hetero-evaluation of the Degree of Competence was performed using the Competence Scale of Actions of Nurses in Emergencies. Finally, after the manager did all the hetero-evaluations, the researcher asked their opinion on who were the most competent, the more or less, and the least competent nurse among the ones evaluated. To evaluate this control group, the manager should use as a reference metric from 1 to 5, according to the Scale of Classification the Degree/Level of Professional Competence. 

As for the Analytical Procedures, because the entire set of data collected during the application of the Instrument created conditions for multi-methods and multi-informants, the instruments were sequentially organized as they were filled by nurses from the Hospital Emergency Services, the AMC unit and, finally, the SAMU unit. The information was entered in Microsoft Excel 2007® worksheets. The errors and missing data were checked with the *Missing Values Analysis* (MVA). The floor and ceiling effects were also analyzed to identify the minimum and maximum values of the responses^12^. 

The analysis of the CSANE was descriptive and inferential. The first allowed describing, summarizing, and obtaining an overview of the data, and the second, through the evaluation of a large dataset, allowed conclusions to be drawn from the study sample and to statistically demonstrate evidence of validity of the Scale. 

Although confirmatory factorial analysis (CFA) is widely used for scales that have evidence of validity previously demonstrated in the literature, this analysis can also be used to verify the plausibility of a theoretical or expected model, based on the researcher’s experience. The CFA corresponds to a particular model of the Structural Equation Modeling (SEM)^12^. 

The CSANE was analyzed for its dimensionality, related to the FCA and exploratory factorial analysis (EFA). The external criterion was also evaluated.

The FCA was used to evaluate the plausibility of the Initial Competence Scale of Actions of Nurses with 81 items, conceptually distributed in eight dimensions: BC1. Care performance (20 items); BC2. Teamwork (13 items); BC3. Leadership (15 items); BC4. Humanization (12 items); BC5. Interpersonal Relationship (10 items); BC6. Decision Making (13 items); BC7. Targeting for Results (14 items); and BC8. Proactivity (15 items). The adequacy of the models was verified through indices such as the RMSEA, CFI, TLI and standardized Chi-Square (X2/d.f.).

As the adequacy indices, after fitting the FCA, did not confirm the theoretical structure, the EFA was carried out in order to evaluate the dimensionality suggested by data in the scale. The EFA was performed through the method of principal components and VARIMAX orthogonal rotation. The criterion for selecting the number of factors was eigenvalue above one. The criteria for exclusion of the items were: values with commonality of less than 0.5 and factorial loads less than 0.5. The Kaiser-Meyer-Olkin (KMO) criterion for the adequacy of the sample and the Bartlett sphericity test were used to evaluate the overall significance of all correlations between the items on the scale considered. 

On the other hand, the psychometric properties of the factors resulting from the EFA were verified through global internal consistency and the sub-dimension was analyzed through the Cronbach’s Alpha coefficient. The Alpha coefficient usually ranges from 0 to 1. The closer to 1, the greater the consistency between items on a scale or subscale.

In the external criterion, the correlation between the hetero-evaluation and the subjective hetero-evaluation about the most competent, the more or less competent, and the least competent nurse was analyzed through the Pearson test, according to the Degree of Competence Scale. We also compared the means of the factors of the hetero-evaluation with the means resulting from the number of most competent, more or less competent, and least competent nurses, according to the manager’s holistic assessment. 

It should be noted that, regardless of the type of factorial analysis used, the scores for both the self- and hetero-evaluation were pooled to generate a single competence structure. Given the fact that, in order to assist a client in an emergency, the nurse must perform excellence actions, as expected for competent professional exercise in emergencies, the competencies assessed through the actions must be the same regardless of whether self- or hetero-evaluation. The EFA was conducted with the information of the two groups of nurses, assuming that the nurse competence construct is unique, detached from the perspective of care provider or management, and therefore, there is no reason to expect differences in the competence evaluation of the different type of evaluator. 

The statistical softwares SPSS 20 and Stata 12.0 were used for psychometric analysis. The SPSS was used for the purpose of descriptive analyses and the *Stata* estimated the FCA models with a significance level of 5%, p < 0.05. 

This research obeyed the national and international norms of ethics in research and was carried out after consent of the Research Ethics Committee under Opinion number 220,513. 

## Results

Two groups of nurses participated: 407 staff nurses and 41 nurse managers, who worked in 11 hospitals from the five regions of Brazil and also in one AMC unit and one SAMU unit, both located in São Paulo. The staff nurses responded to 407 self-evaluations, and the managers, 407 hetero-evaluations, totaling 814 valid protocols with a low percentage of lost cases and errors resulting from typing (0.18%). 

Among the 407 staff nurses, 314 (77.1%) were women and 93 (22.9%) men aged 22-66 years, with a mean age of 36.3 and Standard Deviation (SD) equal to 8.0, and a median age of 35 years. Of these, 194 (47.8%) were from generation Y, 185 (45.6%) from X, and 27 (6.7%) from *baby boomers*. Regarding the place of work, 376 (92.4%) were from hospitals, 22 (5.4%) from SAMU, and nine from (2.2%) AMC. The participants had graduated between 1978 and 2015. As for qualification, 18 (4.4%) had post-graduation *stricto sensu* and 304 (74.7) post-graduation *lato sensu*, and of these, 120 (29.4%) had post-graduation in urgency/emergency. In the last two years, 67.8% took courses in emergency, of whom 36.1% attended courses about protocol for classification of patients. It was verified that 35.1% of the nurses worked in more than one institution.

Among the 41 managers, 32 (78%) were women and nine (22%) men aged 27 to 57 years, with a mean age of 38.7 (SD = 8.5) and median of 36. Of these, 16 (40%) were from generation Y, 19 (47.5%) from X, and five (12.5%) from *baby boomers*. Regarding the place of work, 36 (87%) were from hospitals, four (9.7%) the SAMU, and one (2.4%) from the AMC unit. They had graduated between 1980 and 2011. Regarding qualification, three (7.3%) had post-graduation *stricto sensu* and 35 (85.4%) post-graduation *lato sensu*, of whom 12 (29.2%) had post-graduation in urgency/emergency. In the last two years, 63.4% had attended courses in emergency care, of whom 26.8% in protocol for classification of patients. It was verified that 29.3% worked in more than one institution.

In the distribution of answers to the 81 items of the original instrument, it was noticed that the answer “Not competent at all” did not occur in the self-evaluation except for the items: i (item) 6 “Periodically take part in realistic simulations in emergencies” and i 12 “Make nursing diagnosis for the patient according to the theoretical reference adopted by the institution”. This was different from the findings of the hetero-evaluation. The answer “extremely competent” was given in 70 of the 81 items the self-evaluation, and in 15 of the 81 items of the hetero-evaluation, indicating the presence of a ceiling effect. This happened when more than 15% of the answers were concentrated at the maximum score of the scale^12^ ^,^ ^14^. 

In the initial EFA with the 81 items, seven factors explained 66.1% of the total variance of the items. In the subsequent analyses, the i17, i15 and i13 were eliminated because their factor loads were lower than 0.4 or their commonalities were lower than 0.5. After these exclusions, another EFA was applied, resulting in seven factors that explained 66.5% of the total variance. The choice of this number of factors was based on the number of eigenvalues of the correlation matrix greater than 1, since a small eigenvalue suggests a small contribution of the factor (F) in the explanation of the variation of the original variables. Table 1 presents the analysis of the items.

**Table 1:** Factorial loads, eigenvalues, percentage of variance explained and degree of competence. São Paulo, SP, Brazil, 2016

F*	**BC** ^**†**^				**Factorial load**							
				**i** ^**‡**^	**F1** ^**§**^	**F2** ^**||**^	**F3** ^**¶**^	F4**	**F5** ^**††**^	**F6** ^**‡‡**^	**F7** ^**§§**^	**C** ^**||||**^
**F1** ^**§**^	**1**	**6**		**71**	**0.695**	**0.253**	**0.179**	**0.182**	**0.250**	**0.225**	**0.158**	**0.751**
	**1**	**6**	**8**	**73**	**0.690**	**0.289**	**0.155**	**0.168**	**0.220**	**0.238**	**0.172**	**0.667**
	**1**			**21**	**0.635**	**0.325**	**0.234**	**0.190**	**0.266**	**0.154**	**0.145**	**0.630**
	**1**			**70**	**0.630**	**0.375**	**0.193**	**0.185**	**0.287**	**0.109**	**0.069**	**0.746**
	**1**	**6**	**8**	**74**	**0.629**	**0.346**	**0.287**	**0.186**	**0.198**	**0.155**	**0.179**	**0.685**
	**1**			**52**	**0.627**	**0.274**	**0.290**	**0.200**	**0.063**	**0.262**	**0.140**	**0.708**
	**1**	**8**		**27**	**0.622**	**0.366**	**0.254**	**0.224**	**0.119**	**0.130**	**0.027**	**0.698**
	**1**			**62**	**0.620**	**0.272**	**0.072**	**0.226**	**0.197**	**0.271**	**0.115**	**0.727**
	**1**			**25**	**0.615**	**0.435**	**0.187**	**0.191**	**0.149**	**0.155**	**0.158**	**0.684**
	**1**	**4**	**7**	**61**	**0.615**	**0.295**	**0.210**	**0.170**	**0.115**	**0.197**	**0.225**	**0.677**
	**1**			**75**	**0.609**	**0.193**	**0.207**	**0.188**	**0.067**	**0.241**	**0.218**	**0.640**
	**1**			**57**	**0.588**	**0.430**	**0.268**	**0.241**	**0.150**	**0.181**	**0.025**	**0.596**
	**8**			**77**	**0.579**	**0.453**	**0.252**	**0.237**	**0.124**	**0.153**	**0.146**	**0.710**
	**8**			**36**	**0.574**	**0.338**	**0.340**	**0.256**	**0.114**	**0.141**	**0.189**	**0.708**
	**8**			**45**	**0.548**	**0.113**	**0.134**	**0.366**	**0.216**	**0.300**	**0.166**	**0.642**
	**8**			**32**	**0.533**	**0.279**	**0.442**	**0.077**	**0.200**	**0.152**	**0.133**	**0.720**
	**7**			**58**	**0.528**	**0.315**	**0.268**	**0.174**	**0.441**	**0.141**	**0.077**	**0.642**
	**7**			**64**	**0.525**	**0.257**	**0.297**	**0.274**	**0.392**	**0.085**	**0.181**	**0.714**
	**7**			**60**	**0.523**	**0.174**	**0.335**	**0.365**	**0.267**	**0.154**	**0.200**	**0.567**
	**7**			**31**	**0.512**	**0.486**	**0.054**	**0.198**	**0.296**	**0.101**	**0.060**	**0.698**
	**2**			**68**	**0.504**	**0.335**	**0.338**	**0.221**	**0.250**	**0.136**	**0.194**	**0.717**
	**2**	**7**		**67**	**0.502**	**0.233**	**0.431**	**0.211**	**0.020**	**0.198**	**0.317**	**0.693**
	**2**	**7**		**40**	**0.500**	**0.396**	**0.382**	**0.167**	**0.163**	**0.098**	**0.138**	**0.617**
	**6**			**53**	**0.499**	**0.464**	**0.303**	**0.141**	**0.099**	**0.225**	**0.221**	**0.645**
	**6**			**72**	**0.485**	**0.234**	**0.285**	**0.184**	**0.052**	**0.115**	**0.383**	**0.642**
**F1** ^**§**^	**6**			**79**	**0.465**	**0.441**	**0.359**	**0.113**	**0.124**	**0.178**	**0.258**	**0.686**
	**3**			**59**	**0.459**	**0.229**	**0.414**	**0.179**	**0.400**	**0.111**	**0.262**	**0.666**
	**3**			**81**	**0.445**	**0.347**	**0.395**	**0.177**	**0.234**	**0.201**	**0.201**	**0.720**
	**3**	**6**		**34**	**0.434**	**0.354**	**0.316**	**0.328**	**0.354**	**0.139**	**0.148**	**0.675**
	**5**			**44**	**0.434**	**0.243**	**0.353**	**0.296**	**0.024**	**0.299**	**0.259**	**0.648**
	**5**	**8**		**54**	**0.425**	**0.391**	**0.324**	**0.280**	**0.359**	**0.085**	**0.148**	**0.636**
	**4**			**24**	**0.421**	**0.247**	**0.417**	**0.229**	**0.396**	**0.158**	**0.272**	**0.688**
	**4**			**26**	**0.400**	**0.397**	**0.357**	**0.250**	**0.325**	**0.067**	**0.120**	**0.632**
**F2** ^**||**^	**3**			**43**	**0.338**	**0.732**	**0.145**	**0.165**	**0.161**	**0.090**	**0.107**	**0.707**
	**3**			**76**	**0.180**	**0.713**	**0.081**	**0.152**	**0.218**	**0.146**	**0.201**	**0.644**
	**3**			**14**	**0.277**	**0.707**	**0.166**	**0.071**	**0.087**	**0.139**	**0.280**	**0.584**
	**3**	**6**		**66**	**0.269**	**0.684**	**0.159**	**0.198**	**0.236**	**0.154**	**0.150**	**0.645**
	**3**	**4**		**18**	**0.328**	**0.670**	**0.229**	**0.162**	**0.065**	**0.112**	**0.271**	**0.628**
	**3**			**22**	**0.274**	**0.659**	**0.187**	**0.205**	**0.005**	**0.129**	**0.064**	**0.680**
	**3**	**5**		**23**	**0.325**	**0.637**	**0.232**	**0.217**	**0.026**	**0.148**	**0.100**	**0.734**
	**5**			**78**	**0.207**	**0.579**	**0.247**	**0.197**	**0.359**	**0.036**	**0.139**	**0.713**
	**5**			**80**	**0.176**	**0.563**	**0.213**	**0.222**	**0.293**	**0.161**	**0.172**	**0.725**
	**5**			**69**	**0.483**	**0.541**	**0.239**	**0.274**	**0.115**	**0.154**	**-0.035**	**0.696**
	**2**	**4**	**5**	**55**	**0.481**	**0.529**	**0.249**	**0.160**	**0.156**	**0.048**	**0.137**	**0.607**
	**2**	**4**	**5**	**63**	**0.375**	**0.520**	**0.347**	**0.144**	**-0.029**	**0.026**	**0.195**	**0.591**
	**2**			**49**	**0.300**	**0.499**	**0.260**	**0.236**	**0.390**	**0.071**	**0.097**	**0.665**
	**4**			**65**	**0.393**	**0.496**	**0.125**	**0.307**	**0.454**	**0.127**	**0.045**	**0.744**
	**4**			**29**	**0.468**	**0.494**	**0.297**	**0.188**	**0.187**	**0.183**	**0.096**	**0.627**
	**4**			**19**	**0.325**	**0.476**	**0.329**	**0.280**	**0.358**	**0.199**	**0.011**	**0.686**
	**7**			**05**	**0.210**	**0.454**	**0.314**	**0.372**	**0.225**	**0.200**	**0.103**	**0.588**
	**7**	**8**		**02**	**0.421**	**0.441**	**0.150**	**0.373**	**0.364**	**0.056**	**0.108**	**0.643**
	**6**	**7**		**48**	**0.168**	**0.407**	**0.100**	**0.352**	**0.351**	**0.265**	**0.349**	**0.680**
**F3** ^**¶**^	**8**			**37**	**0.308**	**0.189**	**0.581**	**0.201**	**0.264**	**0.244**	**0.190**	**0.674**
	**8**			**20**	**0.149**	**0.127**	**0.576**	**0.206**	**0.222**	**0.449**	**0.042**	**0.666**
	**8**			**51**	**0.279**	**0.225**	**0.569**	**0.218**	**0.064**	**0.227**	**0.226**	**0.651**
	**8**			**16**	**0.341**	**0.329**	**0.562**	**0.155**	**0.125**	**0.259**	**0.066**	**0.594**
	**8**			**50**	**0.268**	**0.310**	**0.527**	**0.312**	**0.103**	**0.146**	**0.223**	**0.653**
	**2**			**38**	**0.201**	**0.208**	**0.513**	**0.209**	**0.259**	**0.336**	**0.153**	**0.624**
	**2**			**56**	**0.448**	**0.206**	**0.473**	**0.100**	**0.063**	**0.309**	**0.060**	**0.580**
	**2**	**4**	**5**	**35**	**0.450**	**0.367**	**0.460**	**0.175**	**0.201**	**0.160**	**0.085**	**0.581**
	**6**			**41**	**0.373**	**0.322**	**0.441**	**0.149**	**0.326**	**0.058**	**0.106**	**0.588**
	**1**			**39**	**0.244**	**0.418**	**0.426**	**0.260**	**0.106**	**-0.033**	**0.303**	**0.607**
F4**	**1**			**11**	**0.295**	**0.251**	**0.321**	**0.691**	**0.099**	**0.178**	**0.137**	**0.791**
	**1**			**09**	**0.333**	**0.349**	**0.213**	**0.656**	**0.212**	**0.128**	**0.141**	**0.790**
	**1**			**10**	**0.325**	**0.267**	**0.317**	**0.648**	**0.087**	**0.227**	**0.143**	**0.776**
	**3**			**08**	**0.204**	**0.404**	**0.289**	**0.563**	**0.167**	**0.178**	**0.144**	**0.685**
F4**	**2**	**4**	**5**	**07**	**0.301**	**0.351**	**0.003**	**0.484**	**0.137**	**0.156**	**0.221**	**0.540**
	**4**			**04**	**0.373**	**0.322**	**0.325**	**0.455**	**0.222**	**0.192**	**0.033**	**0.642**
	**7**	**8**		**01**	**0.318**	**0.238**	**0.186**	**0.414**	**0.276**	**0.309**	**0.266**	**0.606**
**F5** ^**††**^	**6**	**6**		**30**	**0.332**	**0.353**	**0.226**	**0.133**	**0.494**	**0.184**	**0.294**	**0.668**
	**2**	**2**		**28**	**0.418**	**0.402**	**0.314**	**0.196**	**0.452**	**0.096**	**0.104**	**0.698**
**F6** ^**‡‡**^	**1**			**06**	**0.177**	**0.067**	**0.187**	**0.098**	**0.050**	**0.744**	**-0.023**	**0.636**
	**1**			**03**	**0.288**	**0.111**	**0.212**	**0.133**	**0.146**	**0.645**	**0.051**	**0.597**
	**1**			**12**	**0.177**	**0.168**	**0.093**	**0.149**	**-0.025**	**0.630**	**0.201**	**0.528**
	**1**			**33**	**0.294**	**0.186**	**0.361**	**0.121**	**0.358**	**0.455**	**0.121**	**0.616**
**F7** ^**§§**^	**3**			**47**	**0.295**	**0.336**	**0.259**	**0.207**	**0.200**	**0.139**	**0.633**	**0.770**
	**3**			**46**	**0.392**	**0.302**	**0.252**	**0.210**	**0.163**	**0.110**	**0.579**	**0.727**
	**2**			**42**	**0.194**	**0.462**	**0.211**	**0.172**	**0.204**	**0.123**	**0.574**	**0.712**
**Eigenvalues**					**14.47**	**12.27**	**7.61**	**5.65**	**4.44**	**4.03**	**3.40**	
**Percentage of total variance explained**					**18.55**	**15.73**	**9.75**	**7.25**	**5.69**	**5.17**	**4.36**	
**Accumulated percentage of total variance explained**					**18.55**	**34.28**	**44.04**	**51.28**	**56.97**	**62.14**	**66.50**	
**Cronbach’s Alpha**					**0.981**	**0.936**	**0.920**	**0.919**	**0.796**	**0.754**	**0.877**	
**Number of items**					**33**	**19**	**10**	**07**	**02**	**04**	**03**	
**BC Number** ^**†**^					**08**	**06**	**04**	**05**	**02**	**01**	**02**	
**AC number** ^**¶¶**^					**24**	**12**	**08**	**05**	**02**	**02**	**02**	
**Sample number**												
**Degree of competence 1**					**1-33**	**1-19**	**1-10**	**1-7**	**1-2**	**1-4**	**1-3**	
**Degree of competence 2**					**34-66**	**20-38**	**11-20**	**8-14**	**3-4**	**5-8**	**4-6**	
**Degree of competence 3**					**67-99**	**39-57**	**21-30**	**15-21**	**5-6**	**9-12**	**7-9**	
**Degree of competence 4**					**100-132**	**58-76**	**31-40**	**22-28**	**7-8**	**13-16**	**10-12**	
**Degree of competence 5**					**133-165**	**77-95**	**41-50**	**29-35**	**9-10**	**17-20**	**13-15**	
KMO*** = 0.988												
**Bartlett sphericity test - Chi(3.003) = 57,353.75 (p < 0.001)**												

*F - Factor; ^†^BC - Basic Competence; ^‡^i - Item; ^§^F1- Professional practice; F2 - Relationships at work; ^¶^F3 - Positive challenge; **F4 - Targeted action; ^††^F5 - Constructive attitude; ^‡‡^F6 - Professional excellence; ^§§^F7 - Adaptation to change; ^||||^C - Commonality; ^¶¶^AC - Associated Competence; ***KMO-Kaiser-Meyer-Olkin

The results of the factorial analysis can be interpreted through the factorial loads. Each of the “factor loads” represents the measure of correlation between the factor derived from the analysis and the original items, and can be interpreted like the Pearson correlation coefficient. Based on the factorial loads, the theoretical reference^7^ ^-^ ^8^, the analysis of the content of the items by factor (Table 1) and its correspondence in the Basic Competencies (BCs) and Associated Competencies (ACs), the factors were described as follows:

- Factor 1: Professional practice - Having procedures that stem from nursing knowledge learned to perform actions related to the practice of the profession increasingly improved in the technical and scientific context, and even in human relations at work.
- Factor 2: Relationships at work - Having communication skills with people in recognition of their own potentialities and deficiencies, as well as the absence of absolute truths, achieving a better quality of professional life and achieving fruitful results.
- Factor 3: Positive challenge - Having efficient and effective propositions before the difficulties that arise in the daily routine at work for the creation of an optimistic work environment for the proposed and executed solutions that contribute to the practice of the profession.
- Factor 4: Targeted action - Acting effectively to achieve the goals and objectives proposed in the work plans, finding the right solutions to meet the various levels of decisions available in the professional environment.
- Factor 5: Constructive attitude - Being assertive in a constant way in the work environment in recognition of the various possibilities that emerge in the daily routine for more effective results.
- Factor 6: Professional excellence - Having a qualified performance recognized by the labor market to stand out among other professionals in the area by adding value to their daily actions.
- Factor 7: Adaptation to change - Recognizing the changes and acting appropriately with the resources available at work for being able to understand them and generate useful solutions through their new knowledge and technical development.

**Figure 1:**
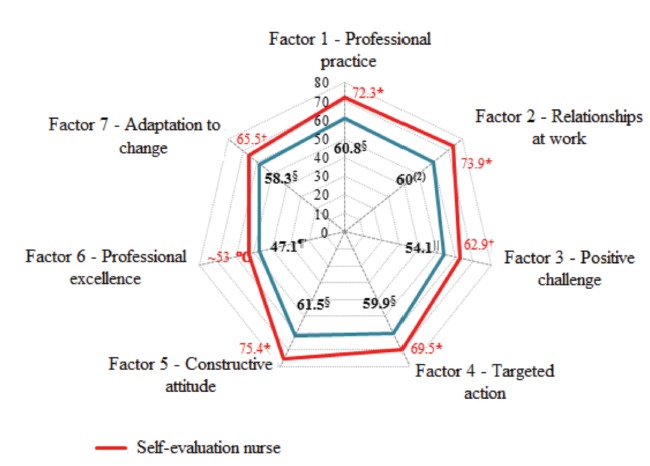
Mean scores by Factor according to type of evaluation. São Paulo, SP, Brazil, 2016

According to Figure 1, the averages of the mean scores of competencies of staff nurses on each factor were higher than those of the managers, indicating that their perception of all aspects is different between these groups and that the first group, i.e. staff nurses considered themselves more competent than their managers believe they are. There is also a difference in the means between factors both in the evaluation of staff nurses (*p* < 0.001) and managers (*p* < 0.001). The means of the factors 1, 2, 4 and 5 were similar among staff nurses, indicating that they see themselves as more competent in Professional practice, Work relationships, Targeted actions and Constructive attitude, more or less competent in the factors Positive challenge and Adaptation to change, and little competent in the factor 6, Professional excellence. This pattern was also evidenced in the managers’ perceptions. That is, the perception of the level of competence according to factors between staff nurses and managers was the same; the only difference is that staff nurses believe to be more competent than what their managers think. 

*Highest mean, ^†^Intermediate mean, ^‡^Lowest mean in the self-evaluation of nurses according to Dunn-Bonferroni multiple comparisons, after Friedman’s test (*p* < 0.001). ^§^Highest mean, ^||^Intermediate mean, ^¶^Lowest mean in the evaluation of managers according to Dunn-Bonferroni multiple comparisons, after Friedman’s test (*p* < 0.001)

To interpret Figure 1, two elements were considered: the area of the figure and its shape. The greater the size of the area, the higher is the mean of the scores in each of the aspects. As for the shape of this area, the more regular the polygon, the more homogeneous is the mean values between the aspects of competencies. Thus, a graph with a very irregular polygon indicates that, on average, the indicators were not similar in the seven aspects, pointing to different levels of competencies.

Table 2 shows the correlations between the factors of the scores in the two groups. In general, these correlations were strong and positive, pointing out that the higher the value of one factor, the greater was the value of the other. There were stronger correlations between the scores of factors in the evaluations of managers than in those of staff nurses. Furthermore, there were weaker correlations between Constructive attitude and Professional excellence and between Professional excellence and Adaptation to change in the view of both for staff nurses and managers. Still, according to Table 2, there were strong correlations between the factors, suggesting the need to evaluate in future studies other non-orthogonal methods of rotation in the EFA. 

**Table 2:** Pearson’s correlation between the factors of the scores in the evaluations of nurses and managers. São Paulo, SP, Brazil, 2016

	**Nurses**						
	**Factor 1**	**Factor 2**	**Factor 3**	**Factor 4**	**Factor 5**	**Factor 6**	**Factor 7**
**Factor 1**	**1**						
**Factor 2**	0.873*	**1**					
**Factor 3**	0.854*	0.775*	**1**				
**Factor 4**	0.744*	0.726*	0.680*	**1**			
**Factor 5**	0.735*	0.717*	0.670*	0.569*	**1**		
**Factor 6**	0.565*	0.502*	0.603*	0.521*	0.443*	**1**	
**Factor 7**	0.678*	0.645*	0.643*	0.550*	0.558*	0.442*	**1**
	**Managers**						
	**Factor 1**	**Factor 2**	**Factor 3**	**Factor 4**	**Factor 5**	**Factor 6**	**Factor 7**
**Factor 1**	**1**						
**Factor 2**	0.888*	**1**					
**Factor 3**	0.890*	0.830*	**1**				
**Factor 4**	0.880*	0.872*	0.847*	**1**			
**Factor 5**	0.828*	0.808*	0.770*	0.762*	**1**		
**Factor 6**	0.714*	0.609*	0.727*	0.667*	0.576*	**1**	
**Factor 7**	0.838*	0.865*	0.777*	0.806*	0.761*	0.544*	**1**

*p<0,001

In Table 3, there were no significant correlations between the self-evaluation and the hetero-evaluation, except for F6, Professional excellence, which although significant (^p^ < 0.001), presented a weak magnitude. Thus, according to the functionality of the score, nurses evaluated themselves better than their managers did; except for Professional excellence, both agree, but in a very inconsistent way. On the other hand, in the evaluation of managers, using the score, significant moderate positive correlations were seen in all factors in their hetero-evaluation with the CSANE and the subjective classification with values from 1 to 5. These two different forms of evaluations of managers correlate and move in the same direction. 

**Table 3:** Pearson and Spearman correlations between scores of competencies in the self-evaluation, hetero-evaluation, and subjective classification of managers. São Paulo, SP, Brazil, 2016

**Factor**	Self-evaluation VS* Hetero-evaluation			Hetero-evaluation VS* Manager classification (subjective)		
	**r** ^**P†**^	**p** ^**‡**^	**N** ^**§**^	**r** ^**S||**^	**p** ^**‡**^	**N** ^**§**^
**Factor 1 - Professional practice**	**0.053**	**0.294**	**397**	**0.609** ^**¶**^	**<0.001**	**128**
**Factor 2 - Relationships at work**	**0.056**	**0.266**	**400**	**0.596** ^**¶**^	**<0.001**	**129**
**Factor 3 - Positive challenge**	**0.005**	**0.918**	**400**	**0.576** ^**¶**^	**<0.001**	**129**
**Factor 4 - Targeted action**	**-0.015**	**0.760**	**402**	**0.591** ^**¶**^	**<0.001**	**129**
**Factor 5 - Constructive attitude**	**0.058**	**0.248**	**405**	**0.547** ^**¶**^	**<0.001**	**129**
**Factor 6 - Professional excellence**	**0.181** ^**¶**^	**<0.001**	**405**	**0.498** ^**¶**^	**<0.001**	**129**
**Factor 7 - Adaptation to change**	**0.062**	**0.210**	**406**	**0.557** ^**¶**^	**<0.001**	**129**

*VS - versus; ^†^r^P^ - Pearson correlation value; ^‡^p - p value; ^§^N - number; ^||^r^s^ - Spearman correlation value; ^¶^ Statistically significant scores for *p* < 0.01.

Table 4 presents the means of each factor by characteristics, according to the type of nurses, in which differences were observed. 

**Table 4:** Summary of measures in the seven factors according to characteristics of staff nurses and managers. São Paulo, SP, Brazil, 2016

	**Self-evaluation**		**Hetero-evaluation**	
	**Mean (SD)**	**p** ^**†**^	**Mean (SD)**	**p** ^**†**^
**Factor 1 - Professional practice**				
**Sex**		**0.002**		**ns** ^**‡**^
**Male**	**76.5 (13.5)**			
**Female**	**71.0 (15.0)**			
**Type of Institution**		**0.027**		**ns** ^**‡**^
**Hospitals**	**71.8 (14.7)**			
**SAMU** ^**§**^	**80.4** ^**A||**^ **(14.3)**			
**AMC** ^**¶**^	**68.8** ^**B**^ ** (15.1)			
**1Specialization** ^**††1‡‡**^		**0.023**		**ns** ^**‡**^
**No**	**74.5 (14.1)**			
**Yes**	**70.6 (14.9)**			
**2Specialization** ^**§1‡‡**^		**0.031**		**ns** ^**‡**^
**No**	**74.1 (14.4)**			
**Yes**	**70.0 (14.4)**			
**BLS** ^**||||2¶¶**^		**0.025**		**ns** ^**‡**^
**No**	**74.8 (14.3)**			
**Yes**	**70.7 (14.9)**			
PALS*** ^**2¶¶**^		**ns** ^**‡**^		**0.011**
**No**			**61.2 (19.0)**	
**Yes**			**73.8 (19.8)**	
**Factor 2 - Relationships at work**				
**Sex**		**0.001**		**ns** ^**‡**^
**Male**	**78.2 (13.4)**			
**Female**	**72.6 (14.7)**			
**ATCN** ^**†††2¶¶**^		**ns** ^**‡**^		**0.045**
**No**			**61.6 (18.6)**	
**Yes**			**52.0 (16.9)**	
**Factor 3 - Positive Challenge**				
**Sex**		**0.004**		**ns** ^**‡**^
**Male**	**66.9 (14.4)**			
**Female**	**61.7 (14.8)**			
**Type of Institution**		**0.025** ^**b‡‡‡**^		**ns** ^**‡**^
**Hospitals**	**62.7 (14.8)**			
**SAMU**	**69.8** ^**A||**^ **(14.7)**			
**AMC** ^**¶**^	**55.0** ^**B**^ ** (11.4)			
**2Specialization** ^**§§1‡‡**^		**0.042**		**ns** ^**‡**^
**No**	**64.3 (13.9)**			
**Yes**	**60.5 (15.5)**			
PALS*** ^**2¶¶**^		**ns** ^**‡**^		**0.030**
**No**			**54.6 (19.2)**	
**Yes**			**65.5 (22.0)**	
**Factor 4 - Targeted action**				
**Sex**		**0.006**		**ns** ^**‡**^
**Male**	**73.3 (14.2)**			
**Female**	**68.5 (16.1)**			
**Type of Institution**		**0.012** ^**b‡‡‡**^		**ns** ^**‡**^
**Hospitals**	**69.0** ^**B**^ ** (15.7)			
**SAMU**	**79.2** ^**A||**^ **(16.2)**			
**AMC** ^**¶**^	**68.8** ^**B**^ ** (14.0)			
**Factor 5 - Constructive Attitude**				
**BLS** ^**||||2¶¶**^		**ns** ^**‡**^		**0.032**
**No**			**60.2 (23.4)**	
**Yes**			**66.2 (21.3)**	
**Factor 6 - Professional excellence**				
**Sex**		**0.010**		**ns** ^**‡**^
**Male**	**57.6 (20.5)**			
**Female**	**51.7 (19.1)**			
**Type of Institution**		**0.023** ^**c§§§**^		**ns** ^**‡**^
**Hospitals**	**52.3** ^**B’||||||**^ **(19.7)**			
**SAMU**	**62.5** ^**A’¶¶¶**^ **(15.7)**			
**AMC** ^**¶**^	**59.7 (13.3)**			
***Lato sensu*** **(conclusion)**		**ns‡**		**0.023**
**No**			**42.5 (20.8)**	
**Yes**			**48.6 (24.3)**	
**2Specialization** ^**§§1‡‡**^		**0.037**		**0.006**
**No**	**54.9 (19.3)**		**50.8 (23.7)**	
**Yes**	**49.5 (20.7)**		**42.1 (25.0)**	
PALS*** ^**2¶¶**^		**0.020** ^**a**^ ****		**0.012** ^**a**^ ****
**No**	**54.3 (18.6)**		**47.8 (23.4)**	
**Yes**	**66.4 (18.8)**		**64.8 (24.5)**	
**ACLS** ^**††††2¶¶**^				**0.005**
**No**			**45.9 (24.1)**	
**Yes**			**54.4 (22.0)**	
**Manchester** ^**‡‡‡‡2¶¶**^				**ns** ^**‡**^
**No**	**57.5 (19.4)**	**0.042**		
**Yes**	**52.8 (18.1)**			
**Graduation year**		**0.013**		**ns** ^**‡**^
**Before NCG** ^**§§§§**^ **(until 2004)**	**49.2 (19.2)**			
**After NCG** ^**§§§§**^	**54.6 (19.7)**			
**Generation**				**0.003** ^**c§§§**^
**Millennium Y (22 to 34 years)**		**ns** ^**‡**^	**48.6** ^**A’¶¶¶**^ **(22.1)**	
**X (35-50 years)**			**47.4** ^**A’¶¶¶**^ **(24.6)**	
*Baby boomers* **(51-69 years)**			**33.3** ^**B’||||||**^ **(23.4)**	
**Factor 7 - Adaptation to change**				
**Sex**		**0.001**		**ns** ^**‡**^
**Male**	**72.0 (20.8)**			
**Female**	**63.6 (20.5)**			
**Type of Institution**		**0.044** ^**c§§§**^		**ns** ^**‡**^
**Hospitals**	**64.9** ^**B’||||||**^ **(20.7)**			
**SAMU**	**76.5** ^**A’¶¶¶**^ **(22.1)**			
**AMC** ^**¶**^	**63.9 (20.0)**			
**Residence** ^**1‡‡**^		**0.015** ^**b‡‡‡**^		**ns** ^**‡**^
**Emergency**	**83.3** ^**A||**^ **(16.7)**			
**1Specialization** ^**††**^	54.2B** (16.9)			
**2Specialization** ^**§§**^	**73.3 (3.7)**			
**ATCN** ^**†††2¶¶**^		**0.008** ^**a**^ ****		**ns** ^**‡**^
**No**	**66.9 (21.1)**			
**Yes**	**52.6 (17.9)**			

*SD - Standard deviation; ^†^p - p value; ^‡^ns - not significant; ^§^SAMU - Mobile Emergency Service; ^||^ A - Highest mean; ^¶^AMC - Ambulatory Medical Care; ** B - Lowest mean; ^||^ A and ** B present distinct means according to Duncan’s multiple comparisons. ^††^1 Specialization - Clinical Nursing and Health Care; ^‡‡^1 - Only for nurses who completed *Lato-Sensu*; ^§§^2 Specialization in nursing and health care in a collective dimension; ^||||^ BLS - Basic Life Support; 2^¶¶^ for nurses who attended courses on emergency care; *** PALM - Pediatric Advanced Life Support; ^†††^ ATCN - Advanced Trauma Care for Nurses; ^‡‡‡^ b - ANOVA; ^§§§^C - Kruskal-Wallis test; ^||||||^B` - Lowest mean; ^¶¶¶^A - Highest mean; ^¶¶¶^A and ^||||||^B` present different means according to Dunn-Bonferroni multiple comparisons. **** a - p Descriptive level of the Student’s t-test or Mann-Whitney test; ^††††^ACLS - Advanced Cardiovascular Life Support; ^§§§§^NCG - National Curricular Guidelines

## Discussion

The descriptive analysis showed that less than a quarter of the nurses had participated in realistic simulations (i6) in the last two years, indicating a low percentage of training in scenarios with skill stations, using mannequins and real or fictitious cases. This information is worrying and strange, because the services studied are references of the SUS, which has a policy on continued training in urgency/emergency services. Another item that called attention was the i12 “Make nursing diagnosis...”, because it was expected that nursing diagnoses were done by nurses at the very moment of the consultation to clients, taking into account that the nursing diagnosis is fundamental to direct the subsequent steps of the Systematization of Nursing Care (SNC). Moreover, when nurses do the Nursing Diagnosis (ND) at the moment of classification, they optimize the care in emergency services, favor decision making and prioritize the care of clients soon after their arrival, thus reducing the negative effects of delayed care on the prognoses^15^. Brazilian studies present numerous nursing care actions, but there is no mention of the SNC^16^, the ND is only identified from the analysis of other nurses’ records^15^ ^,^ ^17^ ^-^ ^18^ and although they consider it essential to perform the SNC. The ND is a step of the SNC this process, but nurses find it difficult to do it because of lack of time, high client demand and circulation, insufficient theoretical knowledge and resistance of the nurses themselves to do it^19^. These results raise concerns about what is happening because the SNC is the foundation and professional identity for excellence in the practice.

In the inferential analysis, an attempt was made to reproduce the structure theoretically thought through the FCA in order to evaluate the evidence of validity based on the internal structure, internal consistency and external criteria of the CSANE. By means of a poor fit, the structure of the data was identified with the EFA, in which the proposed items were separated into seven factors. Their explanation of the total data variance was good and evidenced the validity based on the internal structure of the CSANE^12^ ^-^ ^13^.

The EFA showed that there was a difference between the proposed theoretical construction and the practical reality, indicated by the low correlation identified between three items in the grouping according to factors. In relation to item i17 “Form bonds with colleagues at work”, the experience of the authors led them to consider the possibility that this bond is not very observable in practice. In the i15, “Make choices compatible with the freedom of action that you have”, although most nurses have freedom of action in nursing, they feel constrained or do not believe they are autonomous. In the i13, “Clearly perceive the potential of people at work”, it was understood that the nurses do not perceive the potential of each other, including those in the leadership, a position in which it is essential to influence others. An item is deleted when everyone does it or when nobody does it. Given the results, we opted to exclude them.

When designing the logistic regression model for this scale, it was considered the possibility to measure the degree/level of competence of nurses described by actions and data related to their latent trait. For this, the EFA was carried out through the principal components method, but not principal components analysis^20^. The psychometric property of Cronbach’s alpha demonstrated that, among the seven factors, there is internal consistency in 78 items, and that four factors had values very close to 1, indicating good internal consistency.

These results indicate that the dimensional structure is internally consistent, because there was a difference in the pattern of responses obtained among nurses because they had these different levels of latent constructs, supposedly measured by the Scale. Thus, although the theoretical structure thought with 81 items was reduced to 78, these results could be arranged in a dimensional structure with seven factors, which explain its grouping. This one-dimensional structure contemplated all eight BCs and 32 ACs of the PCM originally proposed. 

Based on these data, a transposition of theory to reality was carried out, the result of which confirmed that the PCM is the essential framework to originate the actions, since it suggests the nature of the items used to interpret the factors generated by the EFA, derived from the proposed competencies. It was also possible to affirm that, although this factorial result has a theoretical dimension of its own, the interdependence and interrelationship of the competences created in the PCM were maintained and confirmed because a correlation was demonstrated in the reallocation of competencies by factor. 

The EFA found that, as in theory, although BCs are different, they are so interrelated that it is difficult to generate a single pure factor with only one type of Basic Competence (CB). Analysis with BCs and ACs allowed a more detailed understanding of each item/factor, since these are grouped as a result of their correlations with all the others. The relevance of the items in the Scale, plus their combination according to the factorial load, made their structure differ from the theoretical PCM conceived. These inferences occurred after the conceptualization and analysis of each theoretical factorial dimension, from the recomposition of the matrix and its respective items in a large map of meanings.

Research carried out abroad on the validation of an instrument for the evaluation of the competence of nurses had psychometric values close to those of the present study^21^ ^-^ ^23^. 

In this research, the authors did not find a statistically significant difference with respect to the level of competence in the correlation analyses between the self- and hetero-evaluation, i.e. the evaluation made by staff nurses and the one made by managers. The former attributed a higher level of competence than the latter. This can be due to different perceptions of people in the evaluation process and because there are limitations. In the self-evaluation, although it is a moment of introspection, people may find that there is a negative implication in their answers and they may tend overestimate the level of competence, both for themselves and for their visibility. In turn, in the hetero-evaluation, the manager is an observer of the other, and therefore the tendency of overestimation decreases, although it can occur with respect to the favorite workers of the manager. Although this is a disparate evaluation, it is commonly observed in studies of this nature. 

In international surveys containing self and hetero-evaluation, the nurses were better evaluated by themselves than by their managers, corroborating the results of the present investigation. In a comparative study carried out among 330 staff nurses and their respective 19 managers, there was a statistically significant difference between self- and hetero-evaluation regarding the Clinical Competence Scale (NCS)^23^. In another research published in 2015, a matrix built in Sweden was translated and culturally adapted to Norway. The authors used statistical tools such as EFA and principal components analysis and found that it is difficult to know the degree to which the competence attributed by the nurses in their self-evaluation is related to their actual behavior^24^. In a comparative hospital study were 81 self-evaluations and their respective hetero-evaluations were conducted, it was identified that the level of competence is related to the intensity of the practice of such competence^25^.

In addition to the comparisons between self- and hetero-evaluation, the authors compared the evaluation of managers through two different methods. The degree of competence attributed by the managers using the CSANE was compared to a subjective parallel evaluation in which they orally attributed a score from 1 to 5 to the most competent, the more or less competent, and least competent nurse. There was a significant correlation among all the hetero-evaluated, that is, the two different forms applied resulted in a consistent evaluation of managers. These data show that the CSANE converges with the manager’s subjective assessment, since it is a relevant external variable associated with the evaluated construct. Therefore, they demonstrate evidence based on external criteria. 

In short, there was no correlation between the self-evaluation and the hetero-evaluation, with the exception of Professional excellence. This demonstrated that managers are more rigorous than the staff nurses. In turn, the hetero-evaluation using the CSANE and the subjective hetero-evaluation of managers were correlated, indicating that it is possible to qualify in which aspect the nurse is more competent through the CSANE.

In addition to assessing the CSANE structure through dimensionality, its sensitivity to observable variables such as personal and professional profile and continued education related to improvement were also considered.

Male staff nurses considered themselves more competent in all factors, while managers see no distinction between genders. As for age, there was no difference between the three generations when self-assessed. However, there was a small numerical difference among *baby boomers*, who considered themselves more competent than others. On the other hand, the managers indicated a greater professional excellence for staff nurses of generation X and Y. In a study carried out with 2052 Finnish nurses, there were significant differences between these three generations with respect to competencies^26^.

Regarding graduation, staff nurses who completed the undergraduate course after the implementation of the National Curricular Guidelines were judged to be more competent in “Professional excellence” than those who graduated before that. This may have occurred because competencies have guided the training process.

Regarding the place of work, the assistants who worked in the SAMU considered themselves more competent, while those who worked in the AMC unit believed to be less competent. This data was not evidenced in the hetero-evaluations of the managers. It is inferred that the fact that SAMU care is perceived to be more competent is because this service is associated with greater freedom during the care to safeguard the lives of people in diverse environments, and at the same time has limited resources, making them to face challenges very often. A study with SAMU nurses in São Paulo to verify the opinion about the theoretical knowledge and skills required in the professional exercise, among 23 items related to theoretical content and skills, 21 (91.30%) were considered basic or necessary for their professional practice in Mobile PHC^27^ ^).^ In another research, it was affirmed that the nurses must have a solid training in basic areas for action, clinical experience and good perception for early detection of changes that occur in traumatized patients^18^.

As for post-graduation *lato sensu*, the staff nurses who specialized in the modality of residence considered themselves more competent in the factor “Adaptation to change”. They control emotions in the face of adversity and changes in daily work and adapt quickly to unexpected situations without going beyond mental and physical limits when compared to those who have other types of specialization. This data is associated with residence in emergency services because of the intense program of comprehensive development with practical and theoretical-practical activities^28^, with laboratory simulations in different scenarios, situations and complexities, focusing on human and psychomotor relationships. The use of simulations in the laboratory is an advantageous ally in the preparation of the professional’s emotions^29^.

With regard to specialization, the specialist staff nurses were considered more competent by managers in the factor “Professional excellence”. This Excellence was also pointed out by managers in the case of nurses who provided Advanced Life Support (SAV) in Cardiology, or SAV in Pediatrics, compared to those who did not shared in simulations. Therefore, the nurses who continually update their knowledge have a higher quality in the delivery of care.

In short, the summary measures of the seven factors according to the characteristics showed that the nurses trained after NCG, specialists, nurses who updated their knowledge in courses with realistic simulations, and nurses from the SAMU were evaluated as the most competent.

With all these elements that make up the CSANE, it is possible to have a vision of the set of attributes/traits that each nurse has or needs to develop.

Although all the hypotheses were confirmed, this research had the following limitations: firstly, it was impossible to compare it with other similar scales validated in Brazil, because such scales are absent. Secondly, since the sample was of national extent and the collection was made by a single researcher, it was not possible to perform the test-retest, because this would be done with the same sample and within a short time. According to the literature, the test-retest is a form of reliability through which consistency is measured. However, the most commonly used measure to verify reliability is the internal consistency^12^. Considering the above, internal consistency was the measured adopted in this study, which was indicated by the high KMO, showing that the items in the proposed scale measure the construct to which they were designed, and they are interrelated. In addition there were items with maximum mean values of degree of competence. However, care was taken to interpret the data set, seeking meaning to the answers given by the subjects in such a way that they could provide the truest possible information when combined about the nurses evaluated. The scale may need some more specific adjustments according to the point of attention in the UEN, because the PHCSs in this research were all located in São Paulo. It is recommended that in future studies, the FCA be fitted based on the result of the fit of the EFA, but in another sample, as recommended.

It should be noted that the CSANE not only has the model of theoretical opinions and elaborations, but it is also based on an empirical study with different evidences of validity.

The CSANE can be used to assess staff nurses both through self-evaluation and hetero-evaluation, as well as to diagnose, monitor, evaluate and plan their scientific-technical development. It is believed that the methodology adopted to create a Measurement Scale based on a Matrix and a Profile may result in the development of new technologies such as the CSANE.

## Conclusion

The statistical procedures performed allowed the conclusion that the proposed CSANE scale, based on the PCM and the PCP, presents evidence of validity based on internal structure, internal consistency and external criteria. The instrument can be considered reliable and valid to measure the real professional competence of staff nurses in Brazilian emergency units.
